# Extracellular vesicle-based biovectors in chronic wound healing: Biogenesis and delivery approaches

**DOI:** 10.1016/j.omtn.2023.05.002

**Published:** 2023-05-09

**Authors:** Garima, Deepika Sharma, Arun Kumar, Ebrahim Mostafavi

**Affiliations:** 1 Chitkara College of Pharmacy, Chitkara University, Rajpura, Punjab, India; 2 M.M. College of Pharmacy, Maharishi Markandeshwar (Deemed to Be University), Mullana-Ambala, Haryana 133207, India; 3 Department of Pharmaceutical Sciences, School of Health Sciences and Technology, UPES, Dehradun, India; 4 Department of Pharmacy, School of Health Sciences, Central University of South Bihar, Gaya 824209, India; 5 Department of Medicine, Stanford University School of Medicine, Stanford, CA 94305, USA

**Keywords:** MT: Special Issue - Exploiting Extracellular Vesicles as Therapeutic Agents, extracellular vesicles, stem cells, chronic wound, biomaterials, nanomaterials, inflammation

## Abstract

Chronic wounds remain an unresolved medical issue because of major social and therapeutic repercussions that require extensive focus. Recent related theragnostic focuses only on wound management and is not effectively promoting chronic wound healing. The rising number of patients with either under-healing or over-healing wounds highlights the ineffectiveness of current wound-healing treatments, and thus, there is an unmet need to focus on alternative treatments. To cover this gap, extracellular vesicles (EVs), for targeted delivery of therapeutics, are emerging as a potential therapy to treat both acute and persistent wounds. To address these issues, we explore the core biology of EVs, associated pharmacology, comprehension of immunogenic outcomes, and potential for long-term wound treatment with improved effectiveness and their nonacceptable side effects. Additionally, the therapeutic role of EVs in severe wound infections through biogenetic moderation, in combination with biomaterials (functional in nature), as well as drug carriers that can offer opportunities for the development of new treatments for this long-term condition, are also carefully elaborated, with an emphasis on biomaterial-based drug delivery systems. It is observed that exploring difficulties and potential outcomes of clinical translation of EV-based therapeutics for wound management has the potential to be adopted as a future therapy.

## Graphical abstract

**Figure:**
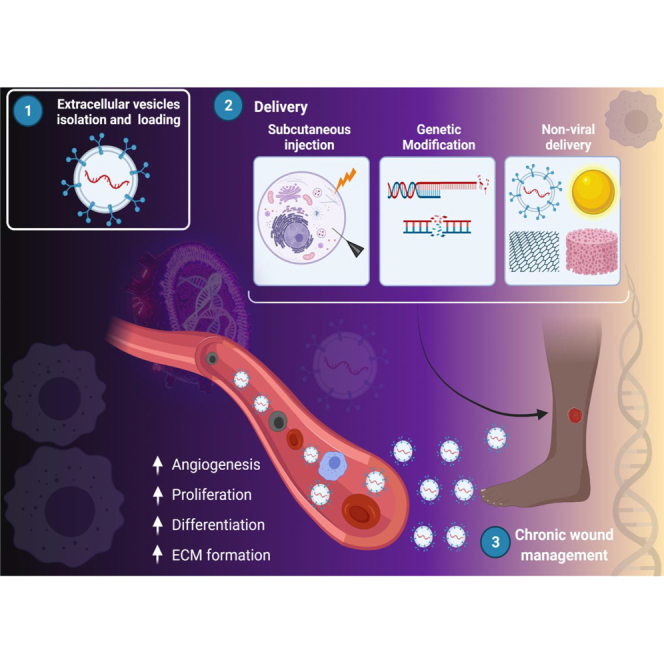

## Teaser

Mostafavi and colleagues explain that a chronic wound tends to remain an unresolved medical issue, which is due mostly to social and therapeutic repercussions. To fulfill this transition, extracellular vesicles (EVs) are recommended in targeted delivery as beneficial therapy in wounds (acute and persistent).

## Introduction

Chronic ulcers are characterized by slow recovery of the epidermis and adjacent tissues that lasts more than three months.^1^ These ulcers have created a substantial challenge in the health sector, resulting in financial and emotional distress and poor quality of life for ∼2.5% of the total population.^2^ According to the wound healing organization, 15% of senior individuals in the United States have chronic wounds, mostly variable ulcers like vascular pressure, stagnant, and diabetic ulcers.^3^ Impaired wound repair is among the most serious clinical problems, as it increases the risk for gangrene, disability, and even fatality.^4^ Because of the increasing prevalence of the mentioned issue, chronic ulcers account for the highest therapeutic expense amidst of all mammalian dermal disorders, nearing ∼$20,000 million in the United States alone.^5^

In the past few decades, chronic wound care has evolved into its own specialty, with doctors frequently using cutting-edge therapies such as growth factors, extracellular matrices (ECMs), and engineered skin as well as negative pressure wound therapy (NPWT). However, there is still no perfect therapy for chronic ulcers because of the expense, intensive care needs, microbial infections, and poor implementation of therapies.^6^

A recent trend in wound healing field is inclined toward therapies based on extracellular vesicles (EVs). EVs are naturally produced small vesicles by cells that aid in intercellular signal transduction by carrying a variety of bioactive molecules such as lipids, metabolites, proteins, nucleic acids, and others.^7^ The structural similarity of EVs to liposomes makes them ideal for the transportation of medicines. EVs are brand-new prospects for drug delivery systems with excellent biocompatibility, low immunogenicity, and high bioavailability. They offer a channel for bioactive substances to be delivered to specific tissues, cells, and organs, as well as a way of intercellular communication. Additionally, EVs have an advantage over liposomes in that they can better imitate the cell membrane because they are created by the cells themselves.^8^ EVs have several advantages, including abundant resources, low immunological rejection, and the ability to be reengineered or combined with other biomaterials.^9^^,^^10^ In the past, various studies have been conducted to investigate the functional properties of EVs in various pathological conditions and to assess their potential utility as therapeutic treatments.^11^^,^^12^ EVs are given mostly by subcutaneous injection to many places surrounding the wound, and only a few studies have used EVs based composites for skin wound healing.^13^ Interestingly, EVs have been shown to accelerate wound healing and tissue regeneration via a variety of biological processes, such as revascularization, cell proliferation, motility, and neo-angiogenesis.^14^^,^^15^

In the present review we explore the biogeny of EVs, their role in the regulation of immunological responses, and their contribution in various stages of wound healing. Furthermore, using biomaterials, nanomaterials, and genetically engineered EVs, we summarize existing understanding concerning EVs’ potential therapeutic application as a cell-free carrier for healing and tissue regeneration. In addition, basic research routes for maximizing the future clinical potentials of EV-based therapies are outlined.

## EVs biogeny and the phases of wound repair

EVs are membrane-enclosed lipid double-layer assemblies with a spherical shape and a size of 40 nm to 5 μm.^16^ Data on EV biogenesis categorize them into three subgroups: apoptotic particles (∼50–5,000 nm) released upon apoptosis, exosomes (Exos; ∼30–150 nm) produced by sorting endosomes, and microvesicles (∼100–1,000 nm) derived directly from the cellular membrane.^17^

Apoptotic bodies are formed by cellular apoptosis and contain DNA fragments and sometimes organelles from the parent cell. The development of apoptotic bodies occurs with the contraction of the cell membrane followed by condensation of cytoplasm to reduce the size. Parallel to this, nuclear chromatin condenses and alters while the plasma membrane degrades and undergoes blebbing^18^; as a consequence, the cell’s content disintegrates into apoptotic bodies, which consist of membrane-enclosed vesicles. Caspase enzymes play an important role in the production of these EVs by stimulating Rho-associated protein kinase 1, which leads to the activation of the myosin regulatory light chains.^19^ This causes actin-myosin sliding and induces contractile activity, as a result, the cytoskeletal framework sheds and an apoptotic body forms. During the formation, the internally present phosphatidylserine residues are shifted toward the periphery, which is typically identified by macrophages and hence disposed of.^20^

Microvesicles, or ectosomes, are the second largest type of vesicle, with a diameter of 100–1,000 nm, formed by the plasma membrane fission and outer branching.^18^ The development process is thought to be an outcome of a complex interplay between membrane lipids redistribution and cytoskeletal protein contraction.^21^ A number of Ca^2+^-dependent enzymes catalyze the formation of microvesicles, such as flippases in transferring lipids from external to internal leaflet and floppases in moving lipids from internal to the external leaflet.^21^ Additionally, the biosynthesis of Exos, the smallest EVs, is thought to be regulated by multiple signaling pathways.^22^ The internalization of the plasma constituents, macromolecules, solutes, and fluids into a transport vesicle marks the onset of Exo formation. Fusion of one or more transport vesicles or with exiting sorting endosome results in the production of early endosomes which in the next step accumulates proteins and develop into late endosomes, which are subjected to lysosomal degradation. As an alternative, the so formed late endosomes develop into multi-vesicular bodies storing and liberating Exos upon their fusion with the cell membrane.^23^

Trauma or surgical incisions can produce acute wounds, but chronic inflammatory or diseased conditions result in recurring wounds that do not heal adequately.^24^^,^^25^ The healing process is composed of complex and dynamic physiological processes that take place at unexpected times inside the wound and tissue.^26^

Hemostasis is characterized by vascular constriction and blood clotting, both of which lead to platelet aggregation and thrombus formation. Platelet-derived EVs are the most frequent in blood circulation and play a critical role during hemostasis modulation blood clotting through activation of. integrin IIb3 with a strong affinity for fibrinogen. Apart from this, these EVs also stimulate the proinflammatory p-selectin for increasing the thrombin production.^27^ The existence of tissue factors in platelet-derived EVs from healthy individuals have the potential to indirectly affect monocytes in a way that promotes clotting and exposes tissue factors to their surface.^28^ It has been shown that EVs from a wide range of sources work together to promote synthesis of fibrin clots and platelet plug formation, and further act as a framework for immune cell recruitment.^29^ Furthermore, when the protective dermal layer is breached, the underlying tissues are exposed to microorganisms, increasing the likelihood of infection.^30^ The proinflammatory M1 phenotype is the first to arrive at the wound, which activates the anti-inflammatory M2 phenotype.^31^

**Figure 1:**
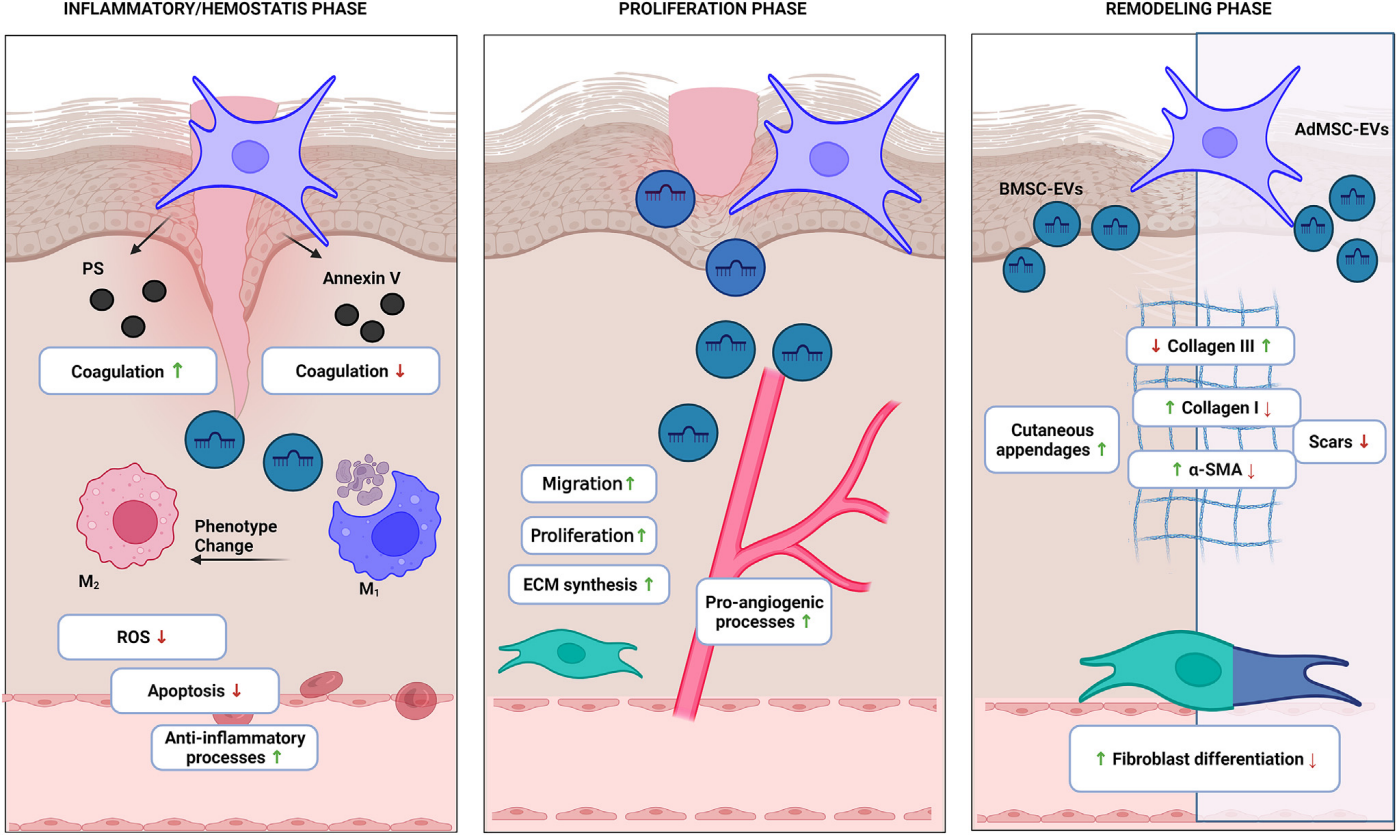
The role of extracellular vesicles (EVs) produced from mesenchymal stem cells in wound healing EVs involve pro-coagulants and anticoagulatory mediators that further regulate hemostasis. Inflammation is aided by EV mesenchymal stem cells by lowering reactive oxygen species (ROS) formation, alleviating apoptosis, and transform proinflammatory phase into anti-inflammatory phase. Furthermore, EVs generated from MSCs stimulate fibroblast migration and proliferation at the site of damage, leading to increased extracellular matrix (ECM) production and promote vascularization. Moreover, mesenchymal stem cell EVs upregulate collagen I synthesis, smooth muscle actin (SMA) synthesis, and progression of fibroblast to myofibroblast but inhibit collagen III synthesis. Furthermore, bone mesenchymal stem cell (BMSC) EVs promote the creation of new cutaneous appendages. Adipose mesenchymal stem cell EVs function in the reverse direction, resulting in scar removal. AdMSC-EVs, adipose mesenchymal stem cell EVs. Reprinted from Narauskaitė et al.^13^ under an open-access license.

The proliferative stage starts after 2–3 days of abrasion and accounts for fibroblasts migration to initiate ECM formation, collagen deposition, epithelization of keratinocyte, and ultimately wound closure (Figure 1; proliferative phase).^32^ All these events suggest that EV-based approaches can be used for the management of long-term inflammatory conditions. At molecular level, it has been reported that EVs derived subcutaneously stimulates numerous signaling pathways by delivering microRNA (miRNA) to fibroblasts and keratinocytes.^12^

In related studies of wound-healing processes, Wnt4-catenin signaling pathway has recently been linked to EVs produced from human umbilical cord blood cells.^33^ Similarly, *in vitro* and *in vivo* studies demonstrate that EVs formed from umbilical cord-derived EPCs provokes angiogenesis in healthy and diabetic wound models. In vascular endothelial cells, they cause the upregulation of pro-angiogenic mediators such as E-selectin, angiopoietin, fibroblast growth factor 1, cyclooxygenase 2, and the cell cycle activator c-Myc.^34^^,^^35^ Of note, the investigators explained that these properties depend mainly upon ERK1/2 signaling and hypothesized that miR-21, which is localized extensively in EVs, activates these signaling pathways.^35^

Remodeling is a time-consuming process and can last anywhere from a few weeks to many months. The remodeling process involves the alteration and remodeling of the ECM as well as scar formation (Figure 1; remodeling phase). During initial; stages of wound healing, type III collagen is prevalent, although it is rapidly replaced by type I collagen, which is the predominant fibril collagen seen in skin. Scarring occurs when myofibroblasts create an excess of collagen and fibroblasts produce an excess of ECM. Surprisingly, in some *in vivo* experimental circumstances, subcutaneous-derived EVs have showed promise in reducing scar formation.^12^^,^^36^ Scar formation has been linked to miRNAs found in the “cargo” of EVs, including as miR-21, miR-23a, miR-125b, and miR-145, suggesting that EVs loaded with such miRNAs might be used to inhibit scar formation.^37^

## EV delivery approaches for cutaneous wound management

Because of their involvement in numerous common signaling pathways, EVs may be engaged in a range of pathogenic reactions.^38^ Many investigations have been conducted to date in attempt to change the expression and distribution patterns of EVs for therapeutic purposes. In this respect, researchers are interested in the effective delivery of EVs alone or in combination with drugs or nucleic acids to enable cell-specific targeting with therapeutic efficacy.^39^^,^^40^ Considering EVs’ fragile nature, isolation procedures are ineffective, and encapsulation efficiency is low, creating and improving efficient delivery systems is a major challenge in EV-based therapies. This section focuses on EV distribution using direct delivery, genetic alterations, biomaterial scaffolds, and targeted delivery of EVs using nanoparticulate approach for wound healing applications.

## Direct delivery and genetic modifications of EVs

Exos can also be delivered directly to wounds via local injection or via topical route. Exo delivery via injection at wound site is mostly preferred but it may be painful and may cause distress to patients. Alternative option is topical application of Exos to wounds, this delivery method not only minimizes the patient discomfort on one hand, but it also helps in delivery of higher dose of Exos directly into wound. However, selection of Exo delivery route depends on type of formulation, severity of wound and patient acceptance; as each route has its own benefits and limitations.^41^^,^^42^^,^^43^^,^^44^ There are multiple studies focused on direct delivery of Exos to wounds. One such study was carried out by Zhang et al. (2018) to promote skin wound healing, this group delivered human adipose-derived mesenchymal stem cell (AD-MSC)-derived Exos directly to the skin wounds of mice, which improved the rate of wound healing and reduced the scar size. Similarly, in a different work, human umbilical cord-derived mesenchymal stem cell (UC-MSC)-derived Exos were injected locally into skin wounds in mouse model. The Exos were found to promote the collagen fiber maturation and accelerate re-epithelialization.^45^ Similar work was done by Jiang et al. (2020), who delivered human BM-MSC-derived Exos locally via injection into skin wounds in mice. Exos reduced the formation of scar along with reduction in production of TGF-β1, collagen I, collagen III, and α-SMA protein.^46^

On the other hand, only a few studies offer insight on the role of direct delivery of Exos in burn wounds. Zhang et al. (2015) described the relationship between inflammation and burn healing. This group introduced human induced pluripotent stem cell (iPSC)-MSC-derived Exos via local injection at the burn site in rats and found that the wound healing effects depended on Exo transfer of Wnt4. Exos not only stimulated the neo-angiogenesis process, but also promoted collagen maturation required for wound healing. In addition, re-epithelialization was accelerated, and scar width was reduced.^47^

Genetic engineering is another strategy for EVs to heal chronic wounds by gene editing using genetic or biochemical procedures.^48^ For genetic modifications, altered EVs are derived from the donor cells via genetic manipulation and a specific pathway can be targeted for promoting wound healing. For instance, adipose-derived Exos are absorbed by fibroblasts, promoting cell movement, proliferation, and formation of collagen in a dose-dependent way, resulting in enhanced chronic wound repair.^49^ Exos derived from adipocytes were also tested for their pharmacological activity against overexpression of nuclear factor in diabetic rat models *in vitro* and *in vivo*. Exos naturally produced by adipose stem cells stimulate endothelial progenitor cell proliferation and angiogenesis in conditions of high glucose levels. This protective effect is enhanced when nuclear factor erythroid 2-related factor 2 is abundantly expressed. *In vivo* wound healing showed a reduction in ulcerated area, increased formation of granulation tissue, increased level of growth factor expression, and reduction in the level of inflammation upon treatment with adipose stem cells. Upon comparison with unmodified EVs, EVs isolated from UC-MSCs with miR-181c overexpression has shown a stronger suppressive effect on the Toll-like receptors 4 pathway in third-degree burn wound model.^50^ Furthermore, treatment of mice with diabetic foot ulcer promoted by injecting BM-MSC EV cells expressing long noncoding RNA (lncRNA) H19 in the tissue that surrounds the lesion. EVs improved cell migration and proliferation and reduced death in fibroblasts.^51^ The investigations revealed that genetically modifying EVs is a useful way for increasing their wound-healing effectiveness.

## Targeted delivery of EVs through nanoparticle approach

EVs extracted from various human mesenchymal stem cells may serve as therapeutic molecules for cell-free therapeutic applications.^52^ They are, however, severely limited in their clinical applicability because of their limited organ-targeting capabilities and therapeutic effects.^53^ Magnetic nanoparticles (NPs), such as ferroso-ferric oxide NPs and ferric oxide particles, have recently gained huge interest because of their prospectives in tissue repair and targeted delivery to the injured site.^54^ With a static magnetic field, they may impose a continuous small magnetic force onto cells to enhance tissue repair.^55^ Furthermore, combining both magnetic NPs and static magnetic fields can improve the healing of wounds by modifying the morphological polarity of fibroblasts.^56^ Magnetic iron oxide NPs are a type of nanomaterial that stands out for its simplicity of fabrication, superconductivity, high magnetism, biocompatibility, and nontoxicity.^57^ NPs are now frequently used in the biomedical area. For instance, they can be created and tailored to function as smart nanobots capable of responding to specific aspects in the extracellular environment to get legitimate, elevated, cell-level, and even genomic pictures of the extracellular environment.^58^^,^^59^ Because of their superconductivity, NPs may be polarized up to magnetization by external electromagnetic guiding yet exhibit no residual magnetic attraction when the magnetic guidance is removed, imparting superior dispersion and targeted abilities.^60^ Furthermore, when the magnetic field strength surpasses the linear rate of blood flow, the NPs are sustained in the desired location.

**Figure 2:**
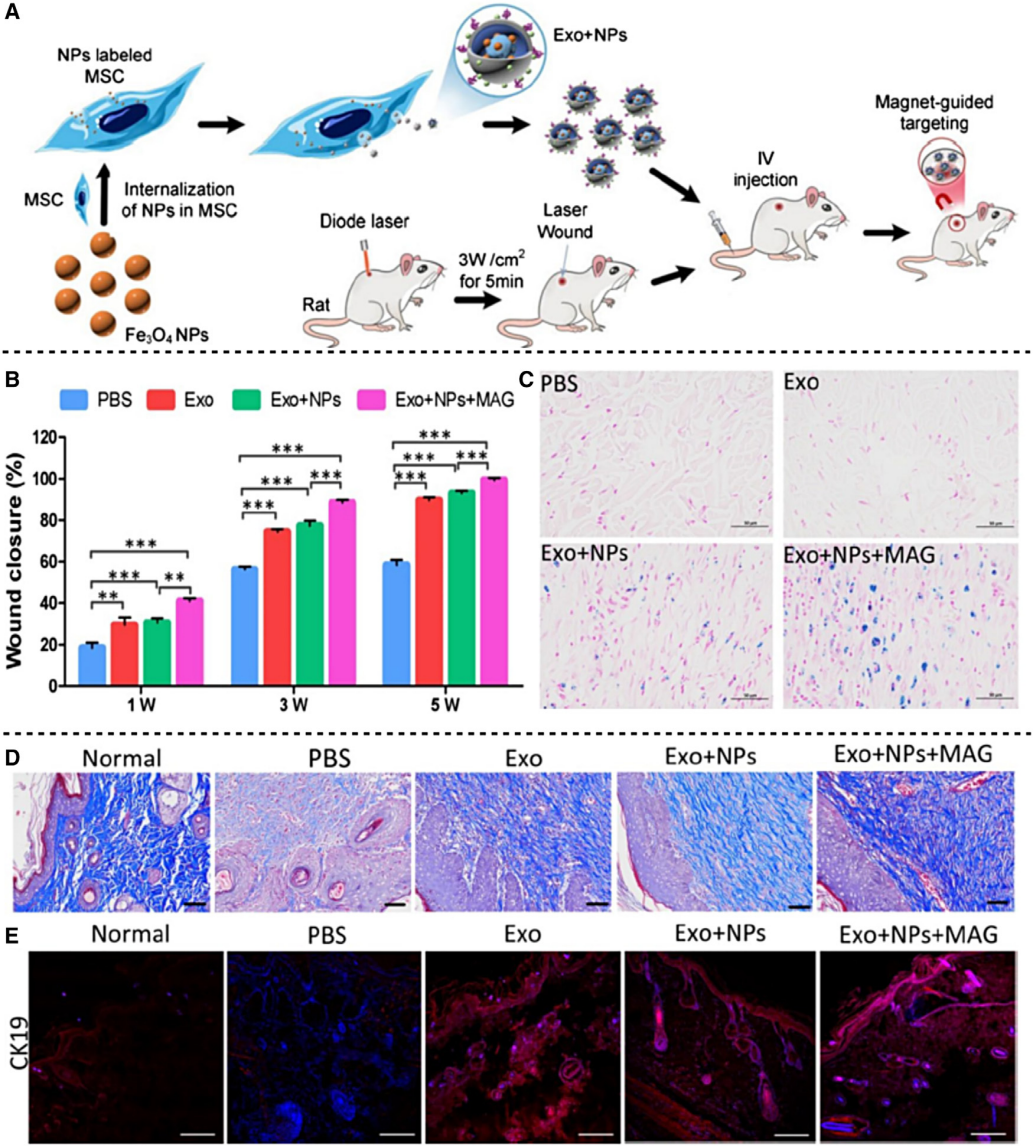
Preparation of magnetic nanoparticle (NP)-labeled exosomes (Exos) and NP-labeled mesenchymal stem cells (MSCs) and *in vivo* targeting to the wounded site (A) Schematic illustration of NP-encapsulated Exos from Fe_3_O_4_ NP-labeled MSCs followed by targeted delivery to the injured skin using the magnet. (B) Graph depicting a quantitative investigation of wound closure rate at various time intervals following wounding. (C) Blue dots showing iron deposition after iron staining in cutaneous wound healing. (D) Photographs showing collagen maturity after staining with Masson’s trichrome 5 weeks after wounding treatment with PBS, Exo, Exo + NPs, and Exo + NPs + Mag. (E) Descriptive fluorescence images showing expression of CK19 in terms of re-epithelization in the wounded area. Reprinted from Li et al.^61^ under an open-access license.

In an intriguing work, researchers isolated Exos from mesenchymal stem cells to improve *in vivo* targeting by manufacturing iron oxide tagged Exos NPs and testing their therapeutic characteristics in an animal model of wound healing (Figure 2A).^61^ The author reported for the first time that strong magnetic guiding can improve the movement of iron oxide labeled Exos and increase the number of NPs that collect at the wounded location, expediting skin wound healing by boosting collagen and new blood vessel creation.^61^ Furthermore, after Exo therapy, it was found that the protein synthesis of cyclin D1 and cyclin A2 as a marker of proliferation, vascular endothelial growth factors, and CXCL12 as a marker of migration was considerably elevated. It was also found that Exos synthesized from untreated mesenchymal stem cells labeled with iron oxide NPs worked as a magnet-guided navigation mechanism. *In vivo*, these accumulated Exos labeled iron oxide NPs significantly increased endothelial cell proliferation and migration, as well as vascular tube formation; second, they reduced scar formation and increased levels of cytokeratin-19, proliferating cell nuclear antigen, vascular endothelial growth factor, and collagen (Figures 2B–2D). All of these observations support the production of therapeutically effective EVs and indicate their potential in epidermal tissue regeneration when targeted with magnetic NPs.

**Figure 3:**
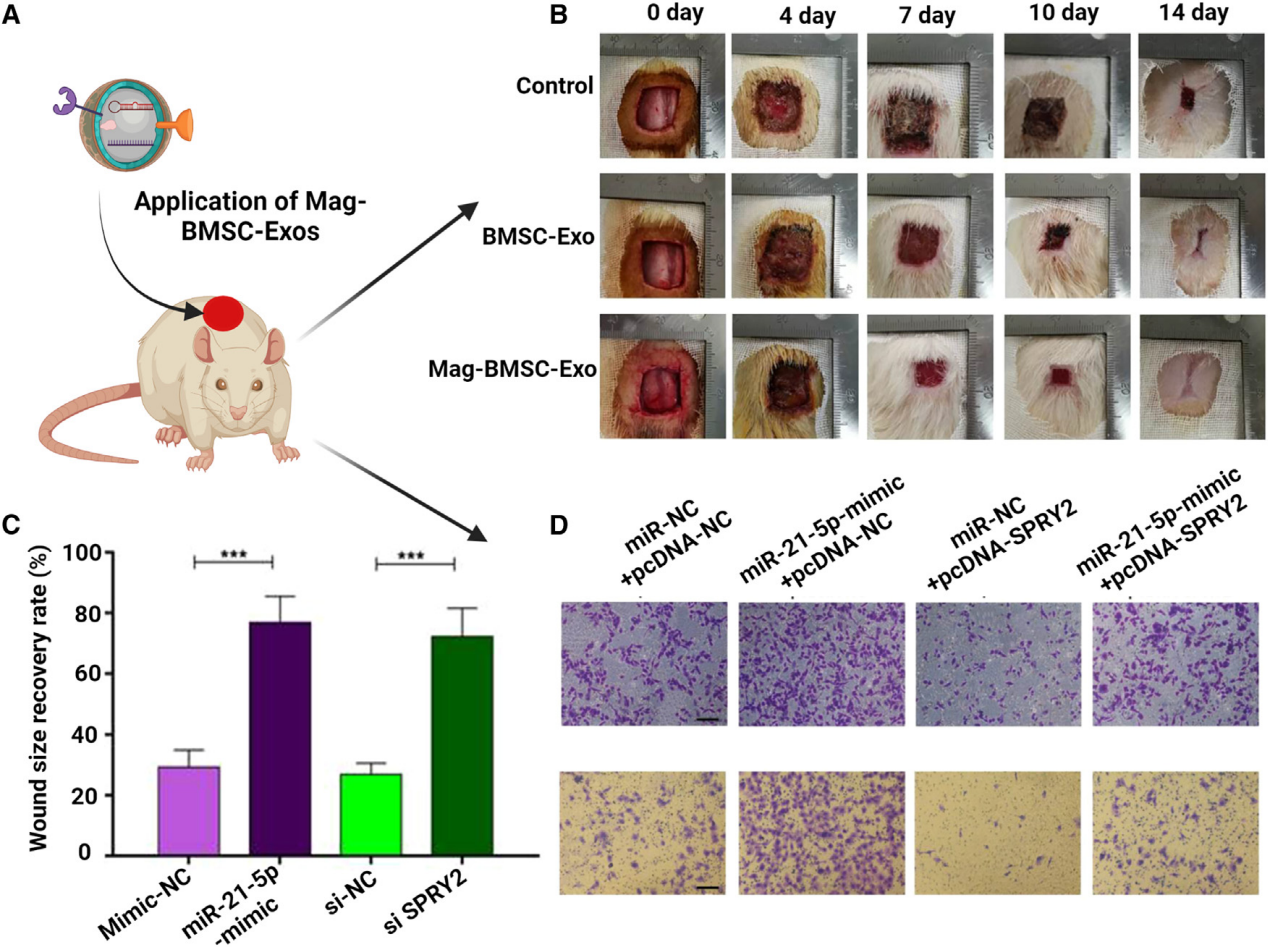
*In vitro*, magnetic exosomal miR-21-5p targets SPRY2 and stimulates angiogenesis and fibrogenesis (A) Schematic representation of how miR-215p targets SPRY2 and activates PI3K/AKT and ERK/2 signaling. (B) A comparison of the wounding results with PBS, BMSC-Exos, and Mag-BMSC-Exos. (C) Graph representing the rate of wound closure at various intervals following wounding. (D) Representative images showing migrated human umbilical vein endothelial cells (HUVECs). Scale bar = 200 μm. Abbreviations: HFS, human skin fibroblasts; AKT, protein kinase B; ERK: extracellular signal-regulated kinase. Reprinted from Wu et al.^63^ under an open-access license.

In another study, researchers found that Exos and magnetic NPs produced from bone mesenchymal stem cells (BMSCs) improved wound closure. The experiment involved the fabrication of Exos by stimulating BMSCs with magnetic NPs and static magnetic fields to promote tissue regeneration.^62^ For the synthesis of Exo-derived magnetic NPs, BMSCs were first isolated by centrifugation and further preconditioned with iron oxide NPs. Afterward, *in vitro,* and *in vivo* experiments were conducted to assess the wound closure efficacy in terms of wound scratch assay, tube formation, *trans* well, and wound healing assay. Outcomes of *in vitro* studies demonstrated that administration of magnetic NP (Mag)-BMSC-Exo promotes proliferation, migration, and emergence of new blood vessels in comparison with administration of BMSC-Exo alone. Local delivery of Mag-BMSC-Exo to the wound site increased wound closure, reduced scar size, and enhanced angiogenesis compared with BMSC-Exo delivery (Figures 3B–3D). Interestingly, miR-21-5p was discovered to be abundant in Mag-BMSC-Exo and to play a significant role in Mag-BMSC-Exo-induced regulatory effects via inhibition and activation of the SPRY2, PI3K/AKT, and ERK1/2 signaling pathways, (Figure 3A). Overall, the results showed that Mag-BMSC-Exo improved angiogenesis, miR-21-5p expression, and fibroblast function compared with BMSC-Exo.

## Localized delivery of EVs using tissue engineered constructs

Because of their great biocompatibility and ability to carry and transport cargos across cells, EVs can act as vector for delivery of therapeutic drugs across cells and prevent their degradation during wound repair. At present, exogenous, and endogenous approaches are used to encapsulate drug molecules and nucleic acids into EVs.^64^^,^^65^ However, several limitations including limited encapsulation efficiency for some biomolecules such as polar drugs and loss in stability profile of EVs across the drug-loading procedure prevails. A few external techniques (e.g., sonication and electroporation) can destroy the peripheral charge of EVs, agglomeration, and potentially destroy the morphology and consistency of EVs.^39^^,^^62^^,^^66^ Furthermore, the encapsulation efficiency following co-incubation with EVs was not sufficient for several big proteins (e.g., catalase).^67^ On either hand, numerous endogenous approaches (e.g., transfection) is ineffective, and EV drug incorporation may be dependent on drug polarity and/or RNA sequence.^9^^,^^39^ Unfortunately, determining how to deliver EVs to skin ulcers and to enhance their physiological activities remains a concern. Because of the high exposure of the wounded area to the surrounding environment, the topical application of EV suspension to the wound site inhibits its biological functions. Furthermore, EVs are rapidly degraded by cells; direct applications make it difficult to keep them in the wound area. EVs must be retained at the wound area via a regulated delivery method to exert their biological effect. The human acellular amniotic membrane is a kind of ECM^68^ found naturally and has been extensively used as a transporter for carrying cells and medications to the targeted site because of its biocompatibility and high porosity.^69^^,^^70^

As a result, a recent study hypothesized that the human acellular amniotic membrane can serve as a vehicle for the extended release of urine-derived stem cell EVs and may enhance the wound healing of urine-derived stem cell EVs in the elderly.^71^ *In vivo* results showed that urine-derived stem cell EVs in the human acellular amniotic membrane may effectively accelerate wound healing by improving cellular senescence and decreasing senescence-associated secretory phenotype production in aged skin ulcers. Urine-derived stem cell EVs were used to grow human dermal fibroblasts *in vitro* to understand the process. The results proved that urine-derived stem cell EVs could regenerated senescent fibroblasts by switching the aging phenotypes of senescent human dermal fibroblasts and effectively lowering senescence-associated secretory phenotype production after triggering the NAD-dependent deacetylase sirtuin-1 pathway. As a result, urine-derived stem cell EVs are effective in improving wound treatment in elderly mice by diminishing cellular senescence.

Indeed, rather than depending on EVs’ endogenous targeting to the appropriate tissues, alternative treatment approaches of therapeutic EVs is its local application. This strategy is attracting attention for tissues having external access to the environment, such as the skin. Transdermal administration of EVs has earlier been shown to promote skin healing^72^^,^^73^ and skin graft viability.^74^ Another possible alternate approach of distributing EVs to a particular structural region in demand of reformative therapy is biomaterial-based application. In comparison with subcutaneous injection, embedding EVs into an appropriate biocompatible material provides the ability to manage EV biodistribution inside the host, EV dosage administration, and EV controlled release from the biomaterial. Several investigations reported hybridizing EVs with a variety of biomaterials, including polymeric hydrogels, films, and spongy scaffolds as a composite wound dressing. Hydrogels have received the greatest attention, probably because of the simplicity with which EVs may be combined with a hydrogel before its actual gelation.^75^ Natural and synthetic polymers such as chitosan, gelatin, sodium alginate, polyethylene glycol, poly (glycolic acid), polyurethane can be used alone or in combination for the delivery of EVs in a non-invasive way to the targeted site, respectively.^76^ Three different techniques can be used for encapsulating EVs in hydrogel systems: direct mixing of EVs with polymer before the addition of EVs, physical addition after hydrogel polymerization, and *in situ* synthesis by adding crosslinker and polymers simultaneously.^77^ Table 1 summarizes different scaffolds loaded with EVs which can be used to stimulate re-epithelization.

**Table 1:** Scaffolds loaded with EVs and their potential healing effects

		EV characteristics			
Scaffold material	EVs source	Size	Surface markers	Therapeutic effects	Reference
Chitosan glycerol hydrogel	Human endometrial stem cell (hEnSC)	40–150 nm	CD63	Angiogenesis, epithelial layer, and formation of granular tissue development were improved.	Nooshabadi et al.^78^
Hyaluronic acid	HUC mononuclear cells	100–130 nm	CD9, 63, 81, tumor necrosis factor 101	Increased vascular density, faster wound healing, and full epithelialization.	Mao et al.^24^
Methylcellulose chitosan hydrogel	Placental MSCs	about 62.5 nm	CD9, 63, 81	Increased migration of fibroblasts, angiogenesis, re-epithelialization. Inhibition of apoptosis.	Wang et al.^79^
Chitosan and silk	Human gingival MSCs	127 nm	CD9, 81	Wound healing was faster, neoepithelialization was more abundant, collagen was deposited, collagen was wavy.	Shi et al.^80^
Pluronic F-127, oxidative hyaluronic acid, poly-€-L-lysine based skin graft	ASCs	60–80 nm	CD9,63,8, Alix	Closure of the wound, formation of new dermal appendages, collagen deposition, and re-epithelialization.	Tao et al.^81^
Human acellular amniotic membrane (hAAM)	AdMSCs	47.7–150 nm	CD9, CD81	Faster ulcer healing, augmented vascularization, increased ECM production, and deposition of collagen.	Xiao et al.^82^
PEI grafted Pluronic F-127 and aldehyde pullulan	Mouse ASCs	not reported	not reported	EV gel group took less time to repair wounds and deposited more collagen.	Wang et al.^83^
Chitosan and silk	Human PRP	not reported	not reported	In the EV-gel group, wounds closed faster, fewer skin ulcers developed, collagen deposition was greater, and vessel density was higher.	Xu et al.^84^
Matrigel	HUC- Wharton’s jelly	30–100 nm	CD81	In comparison with gel alone, EV gel promoted wound closure and collagen deposition.	Bakhtyar et al.^85^
Peptide	Human uMSCs	100–1,000 nm	CD63,81	In comparison with a blank formulation, uMSCs demonstrated full wound closure, less scar formation, greater expression of the growth factor-SMA, and well-arrangement of healed tissue.	Fang et al.^86^
Alginate-based hydrogel	ADSCs	not reported	Akt, ERK, STAT3	Vasculature, scar tissue, re-epithelialization, and granulation tissue formation on the wound surface.	Shafei et al.^44^
Pluronic F-127	hUCMSC-Exo	not reported	CD31, Ki67	Improved exosome ability, improvement in granulation tissue regeneration, and irregular vascular endothelial growth factor expression, potential to improve diabetic wound healing.	Yang et al.^87^
SIS/MBG based hydrogel	BMSC	not reported	CD9, CD63	Promote granulation tissue formation, well-organized collagen fiber deposition, functional new blood vessel growth.	Hu et al.^88^
PVA/alginate nanohydrogel	HUCMSCs	not reported	SMA, SR-B1, CD31, CD29, CD34	Facilitate the proliferation, migration, and angiogenesis of HUVECs and speed up the process of diabetic wound healing.	Zhang et al.^89^
Polyurethane based oxygen releasing scaffolds	ADSCs	200 nm	CD81	Facilitated faster wound closure, enhanced collagen deposition, faster re-epithelialization, and decreased oxidative stress within two weeks.	Shiekh et al.^90^
Gelatin methacryloyl hydrogel	HUVECs	50–140 nm	CD9, CD63, CD81	The *in vivo* results showed accelerated re-epithelialization, promotion of collagen maturity and improvement of angiogenesis.	Zhao et al.^91^

**Figure 4:**
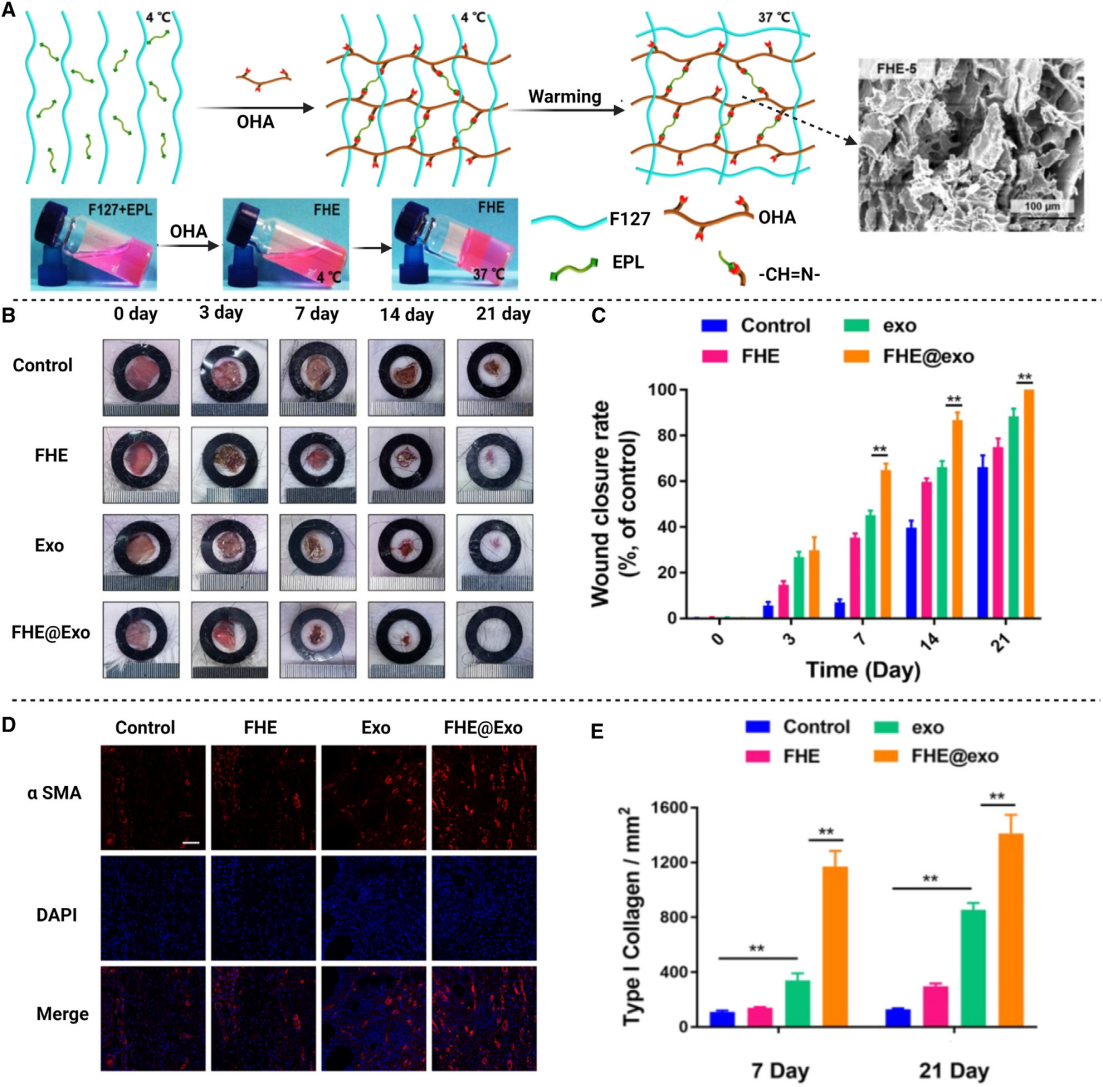
Synthesis and *in vivo* wound-healing activity of injectable hydrogels loaded with Exos (A) Synthesis of thermal-responsive hydrogels using sol-gel techniques composed of F127-EPL and oxidized hyaluronic acid (HA) and optical photographs demonstrating the sol-gel transition of Pluronic F-127/oxidative hyaluronic acid/poly-€-L-lysine (FHE) hydrogel. (B) Photographs showing healing procedure of wounds management using FHE, Exos, FHE@Exos, and control after 0, 3, 7, 14, and 21 days of application. (C) Graph showing closure rates of total wound following 0, 3, 7, 14, and 21 days of application. (D) Blood vessel formation after staining with α-SMA, diamidino-2-phenylindole (DAPI), and merge in wound site 7 days after surgery. (E) Graph representing quantitative assessment of the relative density of type I collagen protein 7 and 21 days after surgery. EPL, poly-€-L-lysine; OHA, oxidative hyaluronic acid; F127, Pluronic F127. Reprinted from Wang et al.^42^ under an open-access license.

A study recently developed injectable, regenerative, stimuli-responsive adipose-derived stem cell Exos and antibacterial polypeptide-based hyaluronic acid (FHE) hydrogels for synergistic wound-healing activity (Figure 4A).^42^ The resulting hydrogel was a pH sensitive gel that delivered EVs in a sustained manner from the porous structure (Figure 4A). During *in vitro* wound-healing activity, FHE/Exo hydrogels have been found to increase the potential of human umbilical vein endothelial cells to proliferate, migrate, and form tubes. Moreover, *in vivo* studies revealed that FHE/Exo hydrogels dramatically improved the healing efficiency in the full-thickness diabetic model, as evidenced by improved wound closure speed, accelerated angiogenesis, re-epithelization, and increase in deposition of collagen at the wound site (Figures 4B–4E). The combination FHE/Exo hydrogel demonstrated better healing effects, showing that prolonged liberation of Exos and FHE hydrogel may both improve diabetic wound closure. Skin tendrils and reduced scarring were also seen in FHE/Exo wound treatment using hydrogel, confirming the gel’s powerful potential to accomplish the full healing process.

Hydrogels containing EVs from various cell origins have already been used to stimulate the healing process. Hydrogels based on polysaccharides, such as chitosan, hyaluronic acid alginate, have already been given clearance by the U.S. Food and Drug Administration (FDA) for wound dressings, and have also been extensively studied for localized application of EVs.^14^^,^^81^^,^^84^ For example, delivery of EVs extracted from platelet-rich plasma, through alginate hydrogels led to an increased wound-healing process and increased angiogenesis compared with platelet-rich plasma hydrogel delivery or hydrogels alone when tested against diabetic wound model during *in vivo* study. Apart from these, *in vitro* analysis confirmed 100% release of EV in 24 h.^14^

**Figure 5:**
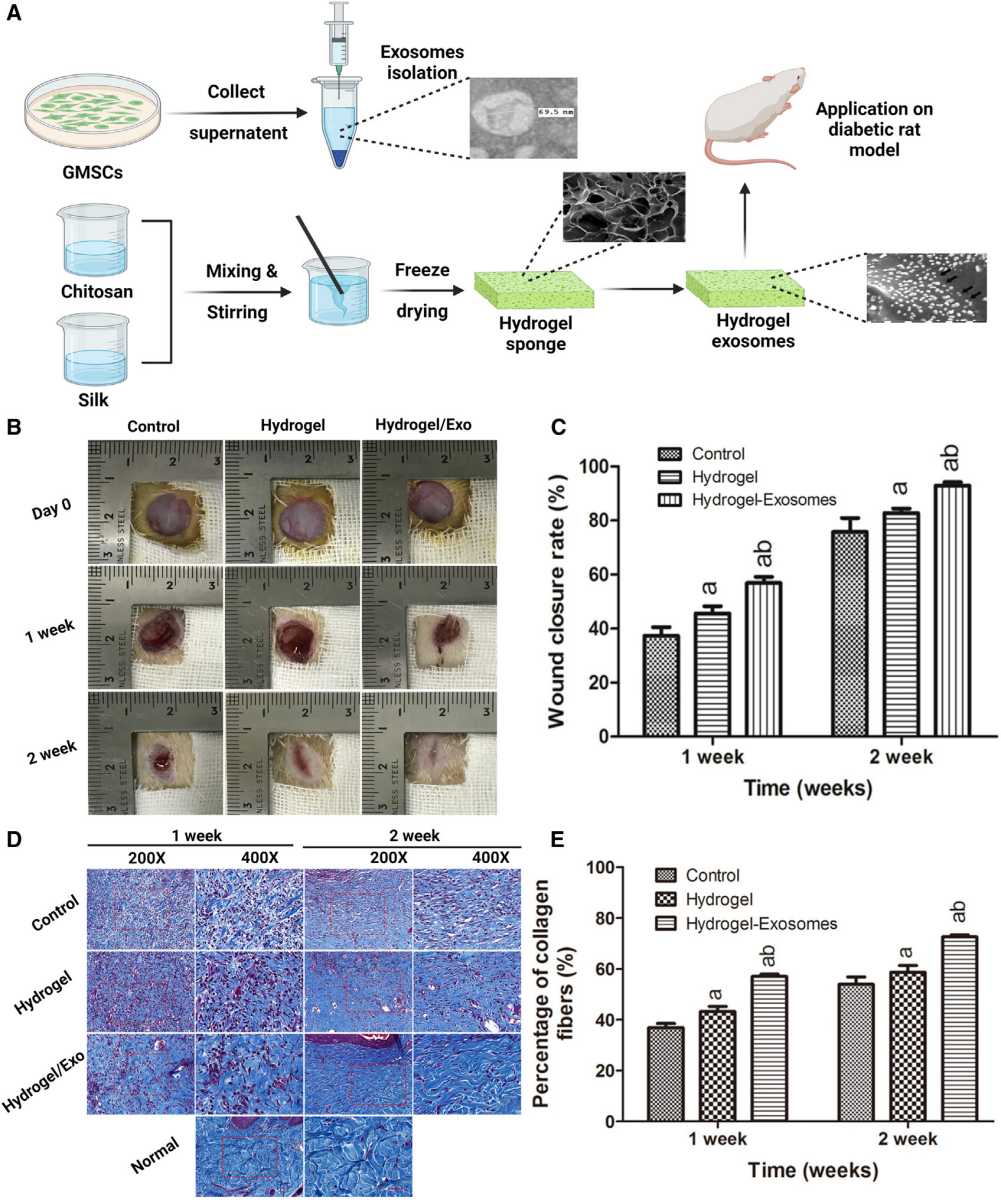
Schematic illustration of chitosan/silk hydrogels loaded with exosomes for diabetic wound healing applications (A) The technique of isolating gingival mesenchymal stem cells (GMSC)-derived exosomes and synthesizing chitosan/silk/exosomes hydrogels for diabetic wound models is depicted schematically. (B) Microscopic photographs of full-thickness diabetic rat model showing wound healing of diabetic rat at 0, 1, and 2 weeks post-surgical procedure in control, hydrogels, and hydrogels loaded with exosome group. (C) Quantitative analysis of wound closure rate at 1 and 2 weeks post-surgery. (D) Photographs showing collagen deposition in wound area after tissues were stained with Masson’s trichrome at 1 and 2 weeks post-surgery. (E) Graphs depicting a quantitative measurement of the proportion of collagen in the control group, hydrogels, and hydrogel/exosomes in each group after 1 and 2 weeks. Reprinted from Shi et al.^80^ under an open-access license.

Similarly, chitosan hydrogel incorporating miR-126-overexpressing synovium MSCs-EVs helped encourage diabetes wound healing, resulting in 15% and 45% release of EVs after 24 h and 6 days, respectively.^66^ Furthermore, chitosan/silk hydrogel sponges were used to treat diabetic foot ulcers^80^ with EVs isolated from gingival mesenchymal stem cells. The isolated EVs showed a particle size of 127 nm and upon loading the silk/chitosan hydrogel had shown an appropriate swelling, porous structure, and moisture retention capacity as an essential property for successful treatment of diabetic ulcers (Figure 5A). The *in vivo* tests demonstrated that EV loading in silk/chitosan hydrogels might significantly prolong wound closure in a diabetic ulcer model (Figure 5B). Histological analysis of silk/chitosan/Exo sponge group had shown additional neo-epithelium and collagen content. Furthermore, the silk/chitosan/Exo sponge group exhibited the highest density of micro-vessels and nerves (Figures 5D and 5E). Matrigel, a commercial product based on cell culture substrate is composed of a combination of ECM-specific proteins, proteoglycans, and growth factors that enable the delivery of EVs for numerous applications, involving healing of wounds. For instance, the application of EVs produced from Wharton’s jelly by Matrigel has been proved to promote cutaneous wound healing in just one week.^85^ Other peptide-based hydrogels are equally widely used to transport EVs for wound repair purposes.^86^

Recently, synthetic polymer has also been used for the delivery of EVs on the basis of hydrogel devices for potential wound healing applications. As an example, for the management of diabetic ulcers, composite hydrogels were combined with either hyaluronic acid and polylysine or Pluronic acid, grafted polyethyleneimine, and aldehyde pullulan.^92^ During *in vitro* investigations, both hydrogel expelled 20% of entrapped EVs in 3 days at pH 7.5 and 80% of EVs in 21 days.^42^ Results also showed that on decreasing the pH to 5.5, the release of EVs was however faster, which contributed to a release of 90%–100% of EVs from both the gels by the 15th day.^42^^,^^83^ In both instances, EV-loaded gels stimulated epidermal renewal and vasculature compared with bare EVs and bare hydrogels.

**Figure 6:**
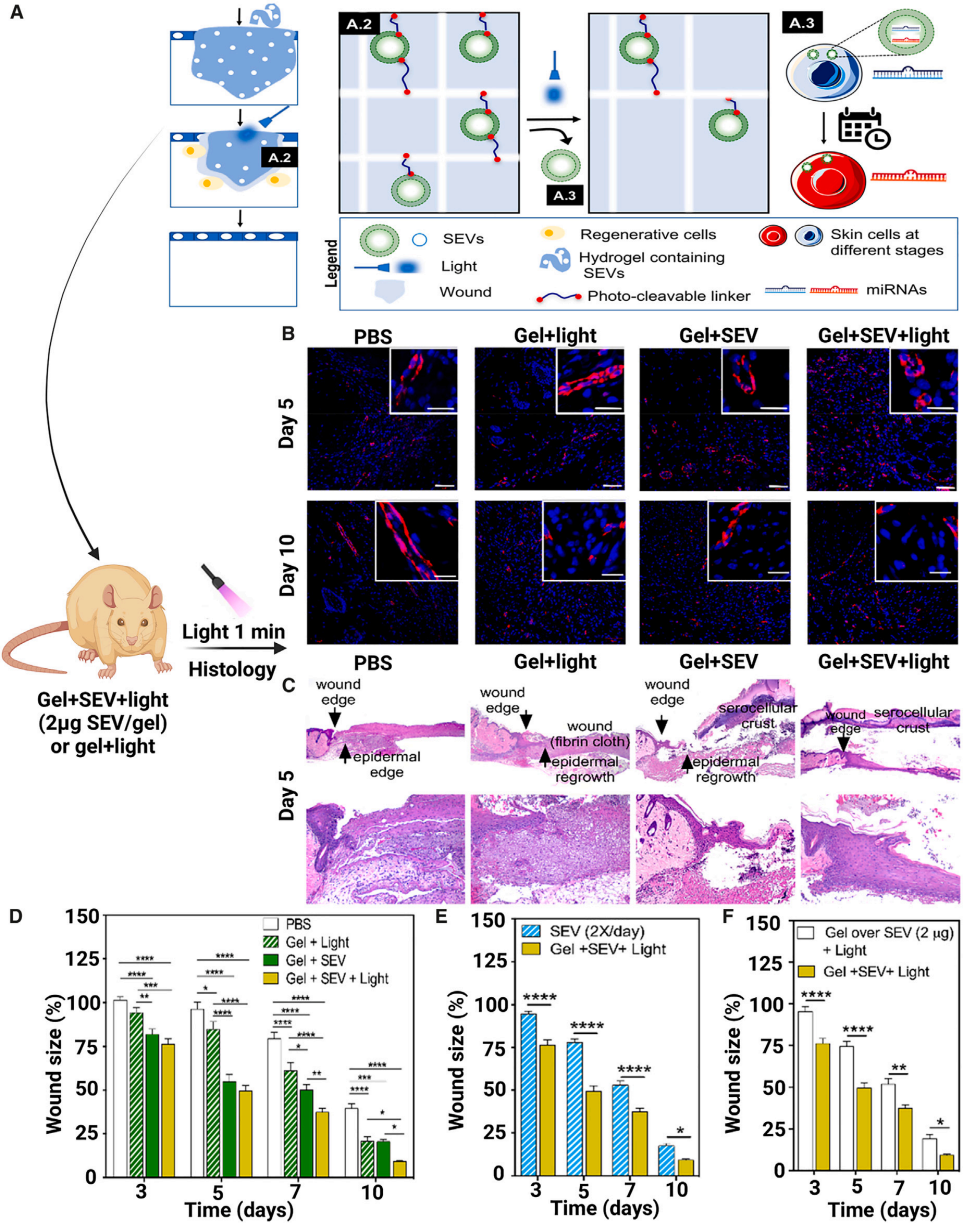
Schematic representation of small EV (SEV) synthesis and topical application of SEV-loaded hydrogels for *in vivo* wound healing potential (A) The proposed role of SEV-containing vesicles and topical application of SEV-containing hydrogels into the wound are depicted schematically (A2 and A3). A light controls SEV release from a distance. Cells enter the space inhabited by the hydrogel as it degrades and are governed by the released SEVs. (B) At day 5, typical photos of H&E-stained tissue slices of implants. (C) Typical CD31 staining pictures taken at five and ten days. (D) The graph depicts type I diabetic wound mice treated with (i) PBS, (ii) light-responsive HA hydrogel exposed every day to a blue laser (405 nm, 1 min for 10 days), and light-responsive HA hydrogel loaded SEVs without (iii) or with (iv) blue laser (405 nm, 1 min for 10 days). (E) Graph demonstrating the percentage of wound region after topical therapy with gel + SEV + light (2 μg EVs per wound) or twice-daily SEV dosages (2 × 0.02 μg day/wound). (F) Graph depicting the percentage of wound region after topical therapy with gel + SEV (2 μg of SEVs per gel; crosslinked with the gel) or SEVs (2 μg) surrounded by a gel (SEVs were not cross-linked). Reprinted from Henriques-Antunes et al.^93^ with permission from the American Chemical Society.

EVs extracted from umbilical cord blood mononuclear cells were connected with a photocleavable crosslinking agent and bonded with hyaluronic acid in a novel technique to make stimuli-responsive hydrogels.^93^ Light irradiation caused the release of EVs from these novel hydrogels, with release rates depending on irradiation time and frequency of irradiations (Figure 6A) This framework has been used to regulate the delivery of EVs in a diabetic wound model for 10 days. Blue laser treatment for 1 min each day resulted in a faster rate of skin regeneration and wound healing than either twice as much EVs alone or platelet-derived growth factor-BB, an FDA-approved wound restorative treatment (Figures 6B and 6C).^93^ Notably, treatment with small EV (SEV)-loaded injectable light-sensitive hydrogels resulted in significantly faster wound closure than SEVs alone or platelet-derived growth factor-BB alone, an FDA-approved wound regeneration remedy (Figure 6D). The tissue/cell phase pro-healing action of released vesicles was achieved by an elevation in epidermal angiogenesis and tissue regeneration, and at the cellular level, there is a shift in the expression of 7 miRNAs during distinct stages of the healing process. This involves alterations in has-miR-150-5p, which was discovered to be essential for skin remodeling.

There are a few more ways that have been used in chronic wound healing, in addition to the several strategies that have already been used and discussed. The text that follows has explanations of these strategies. One of the approaches is three-dimensional (3D) scaffold dressing, constructed out using decellularized small intestinal submucosa (SIS) combined with mesoporous bioactive glass (MBG) and Exos. In addition to enabling the sustained release of bioactive Exos, the produced SIS/MBG@Exos hydrogel 3D scaffold dressing was also observed to improve blood flow and induce angiogenesis in diabetic rats, which hastened diabetic wound healing.^88^

Delivering oxygen is another more recent technique used to heal chronic wounds. It is well established that oxygen administration can control oxidative stress and induce angiogenesis in an infection. OxOBand is an example of a wound dressing based on this method. OxOBand, an antioxidant polyurethane wound dressing containing Exos, is responsible for releasing oxygen. Upon application to a wound, this approach is used to release oxygen and adipose-derived stem cells Exos. In diabetic wounds, the dressing accelerated collagen deposition and re-epithelialization.^90^ In a similar vein, Yuan et al. showed that the synthesis of *in situ* injectable HA@MnO2/FGF-2/Exos hydrogels might speed up the healing of chronic wounds. The finding suggested that upon local injection, these hydrogels were able to create a protective barrier for covering the wounded site with subsequent rapid hemostasis and long-term antibacterial protection. These injectable hydrogels enhanced the angiogenesis and epithelialization in the wound in addition to catalyzing the conversion of excess H_2_O_2_ to O_2_.^94^

Likewise, proper immune responses and pH-responsive-based therapies have also been proposed as a potential and alternative way to facilitate angiogenesis and Exo-based therapy for faster wound healing.^83^^,^^90^ In this regard, an Exo-encapsulated injectable multifunctional hydrogel with self-healing capability was developed by Wang and colleagues with the potential to improve chronic wound healing by releasing stable Exos. This formulated Exo-loaded hydrogel tends to expedite the healing tenure of full-thickness wounds which are caused by diabeties.^90^ On the other hand, using pH-responsive hydrogels promotes angiogenesis and the healing of chronic wounds through pH-responsive Exo release. It has been discovered that FEP@Exos dressing stimulated angiogenesis in diabetic wound tissue and speed up wound-healing process.^83^

## Clinical and pre-clinical status

Because of their extraordinary therapeutic impact and fast development cycle, EV-based natural nanovesicles have become a hot topic in the clinical drug and new drug markets. EVs isolated from numerous stem cells, or comparable related substances, are used in wound healing. According to data, only six clinical trials have been registered for wound healing and related complications; hence, it can be inferred that the transition from the laboratory to the clinic is not as simple as it appears. As a result, further study is required to provide more insight into clinical studies. Products based on stem cells, especially EVs, are controlled and require approval by the FDA. The only FDA-approved stem cell product presently used in the United States is hematopoietic progenitor cells. It was procured from umbilical cord blood for therapy in hospitalized populations with blood production dysregulation. Currently, there are no available FDA-approved products associated with EVs (https://www.fda.gov/vaccines-blood-biologics/consumers-biologics/consumer-alert-regenerative-medicine-products-including-stem-cells-and-exosomes; reviewed on February 16, 2022). ClinicalTrials.gov (reviewed on February 16, 2022) identified six separate conduct of clinical investigation trials using EVs as a treatment for chronic wounds at the time of publishing (Table 2). Two clinical trials were conducted to see if EVs produced from serum may cause a change in wound size and associated inflammation (NCT02565264 and NCT04652531).

**Table 2:** Registered clinical trials with extracellular vesicles in ClinicalTrials.gov

Identifier	Last update	Phase of clinical trial	Condition or disease	Source of EVs	Intervention/treatment	Recruitment status
NCT05243368	February 17, 2022	phase 1	foot, diabetic chronic skin ulcers	mesenchymal stem cells	Dietary supplement: personalized nutritional intervention. Those with malnutrition criteria will also receive a nutritional supplement. The aim will be to provide at least 50% of the recommended intakes for the main nutrients related to wound healing.	not yet recruiting
NCT04134676	June 22, 2020	phase 1	chronic ulcer	Wharton’s jelly mesenchymal stem cells	Drug: conditioned media. These components may be capable of initiating regeneration and repair on their own, as well as mediating the *ex vivo* de novo organogenesis of tissue-engineered organs.	completed
NCT02565264	September 9, 2020	early phase 1	Ulcer	plasma-derived exosomes	The plasma-derived exosomes will be administered to the ulcers of the participants on a regular basis for 28 days.	unknown
NCT04652531	December 7, 2020	early phase 1	Ulcer Venous	autologous extracellular vesicles from serum	The vesicles will be injected into the wound in a sterile environment. An elastic compression bandage and sterile gauze will be used.	recruiting
NCT04281901	August 3, 2021	phase 1	Otitis Media Chronic Temporal Bone	platelet- and extracellular vesicle-rich plasma	Platelet- and extracellular vesicle-rich plasma: ear wick immersed in platelet- and extracellular vesicle-rich plasma	completed
NCT05078385	October 14, 2021	phase 1	Burns	bone marrow-derived mesenchymal stem cells	Drug: AGLE-102. Many of the therapeutic effects induced by MSCs are mediated by EVs derived from MSCs, which may be a safer, more reliable option to allogeneic cell treatment.	not yet recruiting

MSC, mesenchymal stromal cell; AGLE, Aegle Therapeutics.

These findings suggest that the therapeutic advancement of EV-based therapeutics has been slowed because of present obstacles in nanocarriers, a lack of understanding of the mechanism of EVs absorption, and the physiology of multiple pathways.^95^ Several nanocomposites, nanotechnologies, genetic modifications are also being investigated in pre-clinical studies, including scaffolds, liposomes, hydrogels, iron NPs, and Mag. Table 3 highlights the pre-clinical evidence using EVs in cutaneous wound healing.

**Table 3:** Extracellular vesicles in pre-clinical development stage for wound healing applications

EVs source	Model	Inference	Reference
BM-MSC	STZ-induced diabetes	Melatonin-treated EVs derived from MSCs increased wound contraction, Collagen I and III synthesis, and M2 macrophage polarisation; melatonin-EVs enhanced EV impact.	Liu et al.^96^
BM-MSC	STZ-induced diabetic wounds	Wound healing and neo-angiogenesis were enhanced by EVs, with deferoxamine-stimulated-EVs being more effective.	Ding et al.^97^
AD-MSC	STZ-induced diabetic wounds	Endothelial progenitor cells + NRF2-EVs were more effective than EPC + AD-MSC-EVs in promoting wound healing.	Li et al.^49^
BM-MSC AD-MSC	STZ-induced diabetic wounds	Wound healing was accelerated by AD-MSC-EVs but not by BM-MSC-EVs. There was no *in vivo* comparison of scar and vasculature.	Pomatto et al.^43^
UC-MSC	STZ-induced diabetic wounds	EVs promoted wound healing and angiogenesis while increasing VEGF and TGF-1 expression.	Yang et al.^87^
hEnSC	full-thickness excision wound model ischemia-induced wound healing in rats	EVs have enhanced wound closure and high degree of re-epithelialization.	Nooshabadi et al.^78^
HADSCs	ischemia-induced wound healing in rats	In a rat model, CM induced wound closure 50% quicker than unconditioned media and increased cell migration.	Cooper et al.^98^
iPSC	excisional wound splinting in genetic diabetic mice C57BLKS/J leprdb	Enhanced wound closure, decrease inflammatory cells, enhanced skin appendages.	Kobayashi et al.^99^
ESC	full-thickness cutaneous wound-healing model	Enhanced wound closure, enhanced angiogenesis, and decrease scar width.	Hao et al.^56^
HUVECs	full-thickness incisions in STZ-induced diabetic rats	Enhanced re-epithelialization, collagen maturation promotion, and angiogenesis enhancement	Gardiner et al.^100^
ADSC	Full thickness incisions in STZ-induced diabetic rats	EVs showed the requisite proliferative and angiogenic capabilities required to enhance wound healing angiogenesis.	Chen et al.^101^
UC-MSC	full-thickness incisions in STZ-induced diabetic rats	EVs enhanced re-epithelialization and neo-angiogenesis.	Jiang et al.^102^
ADSC	simple murine excisional wound model	Wound healing and neovascularization were both improved by EVs. When EVs were put into a complicated hydrogel, the effect was enhanced.	Li et al.^61^
BM	full-thickness skin wound model in mice	TSG-6 overexpressed MSC-exosomes ameliorate scar pathological harm by lowering inflammatory response and attenuating collagen deposition.	Yang et al.^103^
UC-MSC	deep second-degree burn injury in C57BL mice	EVs accelerated wound healing and angiogenesis, increased expression of VEGF and TGF-β1.	Fan et al.^104^
UC	skin wound-healing model in STZ-induced diabetic rats	Exosome increased endothelial cell proliferation, migration, while decreasing scar formation.	Bakhtyar et al.^85^
UC	skin wound-healing model in STZ-induced diabetic rats	Stimulated *in vivo* angiogenesis by exosome enriched in miR-135b-5p, and miR499a-3p more evidently upon blue light illumination.	Limoni et al.^15^
UC	skin wound-healing model in STZ-induced diabetic rats	Wounds treated with three-dimensional culture-derived conditioned medium heal faster and better than wounds treated with two-dimensional culture. Wounds treated with animal-derived conditioned media.	Bae et al.^105^

BM-MSC, bone marrow-derived mesenchymal stromal cells; AD-MSC, adipose-derived mesenchymal stem cells; UC-MSC, umbilical cord-derived mesenchymal stem cells; hEnSC, human embryonic stem cells; STZ, streptozotocin; HADSCs, human adipose-derived stromal/stem cells; ADSC, adipose tissue-derived stem cells; ESC, embryonic stem cells; iPSC, human induced pluripotent stem cell; HUVECs, human umbilical vein endothelial cells; BM, bone marrow; UC, umbilical cord.

Following clinical investigations along with other regulatory-approved therapies, it is almost certain that not only increased isolation scalability, high purity index, sustained integrity, and functional properties will be needed, but also clear and defined components, availability of standard operating procedures for reproducibility, criteria for quality control, along with sterile environment is of equal importance.^106^ The major topics for investigating EVs will be their transport to the targeted location, their retention in the wound site, the choice between direct administration and the use of a vehicle, low-cost manufacture, immunological tolerance, and harmfulness. Further research into these issues is required to build on the results of clinical trials, which might lead to innovative therapies in the future.

## Challenges in translational and clinical medicines

Overall, the investigations conducted in recent past and details in the current section show that EV either applied locally or systemically offers one of the potential and unique treatment method for wound healing. It’s worth noting that the province has evolved a part from observational study to encompass believable and logical systematic features. Notably, in pre-clinical trials of wound infection, EV-based therapeutics have demonstrated great potential for promoting wound healing and minimizing scarring. As a cell-free adjunct remedy, EVs pose no ethical concerns. Compared with whole cells, EV-based therapeutics offer numerous benefits, including plentiful sources, simplicity of collection, processing, preservation, delivery, and stability.^107^ Furthermore, as they are cell-secreted particles and a route of intracellular interaction, EVs are naturally non-immunogenic. Even if the underlying theory is not fully understood, EV outer membrane proteins or ligands, similarly CD9 and Fas ligand, may lead to EV non-immunogenicity when exposed to the systemic immunological repertoire, as they can prevent improper immune cell stimulation.^108^^,^^109^^,^^110^ However, some challenges in this area must be overcome before the definitive clinical translation of EVs in wound infection can take place.

First, the important challenge is the isolation, standardization, optimization, validation, and purification of EVs. In blood, saliva, urine, and other biological fluids, EVs are often discovered as carriers of cellular information. It is still difficult to extract and isolate these EVs from enormous sources for use in medical field; a variety of sources for use in medical applications. EVs can be extracted and purified using a variety of approaches, including ultracentrifugation, filtration, precipitation separation, immunoaffinity, and microfluidic isolation.^111^^,^^112^ Many of these processes, unfortunately, are time consuming, produce lower yields, and compromise their purity. Several techniques to circumvent these limits are being researched, including cell culture in tangential flow filtering units and bioreactors to increase EV production and size exclusion.^100^^,^^113^^,^^114^

The successful encapsulation of therapeutic cargoes into EVs is the second major challenge for targeted delivery. Therapeutic cargos can be encapsulated into EVs in a variety of ways, similar to lipid-based NPs.^115^ Nevertheless, EV encapsulation effectiveness is lower than that of lipid-based NPs. This could be because EVs integrate some of their own parental cells contents during formation, leaving small space for external drug encapsulation.^116^^,^^117^ However, several drug encapsulation methods have been discovered to date, which can be classified into three broad groups: pre-loading, post-loading, and additional loading methods.^118^ Pre-loading involves encapsulation of cargoes in parental cells using transfection and co-incubation, followed by loading of these parental cells along with cargoes into EVs during their formation process. On the other hand, sonication, extrusion, incubation, and physical approaches like freeze-thaw cycles are examples of post-loading techniques that involve the encapsulation of cargoes directly into EVs. Microfluidics-based and engineering of parental cells are two other loading approaches.^119^

Significantly, there is an utmost need for tissue engineering methodologies to improve the therapeutic efficacy of EVs, like combining EVs with scaffold biopolymers, biological alteration, and encapsulation of EVs with bioactive agents. Furthermore, injecting EV solution into wounds leads to rapid digestion and elimination by the body, limiting EV’s therapeutic efficacy. An innovative technique for the healing of chronic wound that combines both transformed EVs with bioactive molecules (e.g., miRNAs, drugs) and polymeric materials may boost the therapeutic potential of EVs. However, biocompatibility difficulties must be considered carefully while transforming EVs by genetic operations or other chemical approaches. Indeed, it has been claimed that in several diabetic model chronic wounds heal on their own, resulting in variations in results obtained from animal studies and human clinical trials. To properly examine the therapeutic benefits of EVs, standardized animal models are required for chronic nonhealing wounds.

The translation of these nanosystems into clinics poses major challenges in terms of developing a manufacturing method that provides both good quality and quantity. Researchers have worked hard to create good manufacturing practice (GMP)-grade EVs using various ways.^120^^,^^121^ The discovery of a GMP-grade technology for mass-production of EVs from human cardiac progenitor cells was recently revealed by researchers.^122^ Despite all the investment made in studying EV technology, isolation, purification, standardization, developing advanced delivery approaches, and extending its applicability, it has taken longer than projected time to transfer these findings into clinical success, restricting the therapeutic benefit from these efforts. The key causes of EV hurdles in translational medicine include unsatisfactory ideas, deficiencies in isolation and animal models, analytical mistakes, and a lack of clinical relevance.

## Conclusion and future perspectives

A significant amount of research and clinical trials have been conducted over the past few decades on the detection and creation of physiological variables that influence the biological processes that occur during wound healing to improve the healing pace and quality. However, the rising number of patients suffering from persistent wounds or scarring highlights the need for more effective wound-healing treatments. The fundamental disadvantage of EV-based treatment is inadequate isolation, purification, and targeted delivery to the location because of their low stability, and lower encapsulation efficiency because of integration of parental cells. As a result, future research must focus on developing innovative delivery mechanisms with proper isolation and purification techniques for the effective administration of EVs based therapeutics.

Iron oxide NPs, magnetic NPs, cationic polymeric NPs, scaffolds, hydrogels, and even inorganic materials have been found and developed as a carrier for effective delivery of EVs along with cargoes like drugs and nucleic acids. But in addition to the delivery agent, little consideration has been given to the appropriate distribution mechanism. Throughout the phases of the wound-healing process, an effective drug delivery method should discharge the therapeutic agents that match the spatial needs. Several drug delivery technologies that can manage the time-dependent drug release profile, such as passive, active, and smart systems, can be used for EV-based wound therapy. Smart materials that respond to wound biomarkers are examples of smart systems, as are integrated sensing/delivery systems.

Whereas most studies have focused on the use of topical systems, in which the cargoes are delivered directly to the wound bed, there is augmented interest in the delivery of cargoes along with EVs into the deeper layers of the wound using intradermal delivery. The intradermal techniques provide for increased local availability of the EVs in the wound bed by avoiding the complete and robust exudate flow, which is rich in enzymes and can compromise the integrity of the EVs. As a result, future research will likely focus on more efficient EV delivery via intradermal devices.

To conclude, the current findings are encouraging. Although the precise mechanism by which EVs induce wound healing is unknown, EVs have shown beneficial results in promoting chronic wound healing by regulating inflammatory response, enhancing neovascularization, facilitating cell growth, migration, and ECM synthesis, strengthening wound healing mechanisms, and limiting scar formation. Furthermore, EVs can be genetically manipulated, loaded with medicinal compounds, and dressed with biomaterials. Overall, the review highlights how EVs regulate different phases of wound healing and how they can be targeted using tissue engineering approaches as a summarizing remark of this paper. More study is needed to understand the precise actions of EVs in chronic wound healing and to create successful EV engineering methodologies to adapt EV-based treatments for additional therapeutic wound care in the future.
